# Drug Nanocrystals for Active Tumor-Targeted Drug Delivery

**DOI:** 10.3390/pharmaceutics14040797

**Published:** 2022-04-06

**Authors:** Linwei Lu, Qianzhu Xu, Jun Wang, Sunyi Wu, Zimiao Luo, Weiyue Lu

**Affiliations:** 1 Department of Integrative Medicine, Huashan Hospital, Fudan University, Shanghai 200040, China; lw_lu@fudan.edu.cn; 2 Department of Pharmaceutics, School of Pharmacy, Fudan University & Key Laboratory of Smart Drug Delivery (Fudan University), Ministry of Education, Shanghai 201203, China; 19211030077@fudan.edu.cn (Q.X.); wangjun245186@126.com (J.W.); sywu17@fudan.edu.cn (S.W.); luo_zimiao@163.com (Z.L.); 3 Institutes of Integrative Medicine, Fudan University, Shanghai 200040, China; 4 Shanghai Engineering Technology Research Center for Pharmaceutical Intelligent Equipment, and Shanghai Frontiers Science Center for Druggability of Cardiovascular Non-Coding RNA, Institute for Frontier Medical Technology, Shanghai University of Engineering Science, Shanghai 201620, China

**Keywords:** drug nanocrystals, active targeting, drug delivery, cancer

## Abstract

Drug nanocrystals, which are comprised of active pharmaceutical ingredients and only a small amount of essential stabilizers, have the ability to improve the solubility, dissolution and bioavailability of poorly water-soluble drugs; in turn, drug nanocrystal technology can be utilized to develop novel formulations of chemotherapeutic drugs. Compared with passive targeting strategy, active tumor-targeted drug delivery, typically enabled by specific targeting ligands or molecules modified onto the surface of nanomedicines, circumvents the weak and heterogeneous enhanced permeability and retention (EPR) effect in human tumors and overcomes the disadvantages of nonspecific drug distribution, high administration dosage and undesired side effects, thereby contributing to improving the efficacy and safety of conventional nanomedicines for chemotherapy. Continuous efforts have been made in the development of active tumor-targeted drug nanocrystals delivery systems in recent years, most of which are encouraging and also enlightening for further investigation and clinical translation.

## 1. Introduction

According to the Global Cancer Observatory (GLOBOCAN), an estimated 19.3 million newly diagnosed cancer cases and nearly 10.0 million cancer deaths occurred worldwide in 2020, and cancer is not only a leading cause of death, but also a major obstacle to increasing life expectancy in countries all over the world [1]. Chemotherapy is commonly used in the treatment of cancer, yet there exist problems in chemotherapeutic agents such as poor solubility, limited targeting capability as well as toxicity that leads to insufficient drug enrichment at tumor site, thereby hampering their clinical efficacy to some extent [2,3]. With the development of drug delivery technology, different approaches, such as surfactant utilization, inclusion complexation and solid dispersion, have been adopted to prepare safe and efficacious formulations for various diseases [4]. Notably, drug nanocrystals (NCs) are nanosized (particle sizes ranging from 10 to 1000 nm) pure solid drug particles enveloped in a stabilizer layer, which are developed during the crystallization of drug molecules [5,6]. They are a colloidal dispersion system with increased saturation solubility and drug loading due to the large specific surface area and only a small amount of surfactants as stabilizers added [7]. As a mixture of active pharmaceutical ingredients and essential stabilizers, drug NCs with the ability to improve the solubility, dissolution and bioavailability of poorly water-soluble drugs or potential new drug molecules can be administered via oral, intravenous, or other routes [4,5,8,9]. Passive targeting has long been utilized for tumor-targeted drug delivery, but the application of commercial nanomedicines for chemotherapy based on passive targeting such as drug-loaded liposomes, micelles and nanoparticles has hit a bottleneck because of limited clinical benefits resulting from the fact that the enhanced permeability and retention (EPR) effect in solid tumors is weak and heterogeneous in the setting of human tumors [10]. Highly efficient drug delivery at tumor site is attracting increasing attention for overcoming the disadvantages of passive targeting strategy found in practical application, including nonspecific drug distribution, high administration dosage, and undesired side effects [11]. Active tumor-targeted drug delivery, typically enabled by specific targeting ligands or molecules modified onto the surface of nanomedicines, has been greatly validated as a promising strategy to enhance therapeutic efficacy and reduce toxicity ascribed to precise control of drug delivery via specific binding to receptors highly expressed on tumor endothelial cells and/or tumor cells and subsequent receptor-mediated endocytosis [12]. In addition, biomimetic cell membrane coating is an emerging technique with the advantages of both long circulation and active targeting [13]. Continuous efforts have been made in the development of active tumor-targeted drug NCs delivery systems in recent years, most of which are encouraging and also enlightening for further investigation and clinical translation. This review briefly illustrates advances in drug NCs for active tumor-targeted drug delivery, aiming to summarize the current research in this field, discuss the advantages and limitations, and offer perspectives for further studies.

## 2. Preparation and Characterization of Drug Nanocrystals

### 2.1. Preparation Techniques

Compared with conventional pharmaceutical formulations, drug NCs have the advantages of high drug loading, nominal excipient utilization and simple preparation process [14]. Basically, the preparation techniques can be divided into top-down, bottom-up and combination approaches [15].

Top-down approaches, mainly including media milling (NanoCrystal^®^ Technology) and high pressure homogenization (IDD-P^®^ Technology, Dissocubes^®^ Technology and Nanopure^®^ Technology), employ high mechanical force to turn large coarse drug powders into nanosized particles [16]. Top-down approaches are simple and fast, which require no organic solvents and are highly reproducible so that they facilitate scale-up production, so they have been utilized in most commercial products at present (Table 1) [15,16,17,18,19,20]. However, the process is often energy-intensive and time-consuming; high shear and temperature may cause crystal instability and subsequent aggregation. There are also concerns about the probability of product contamination by the grinding media [15]. Bottom-up approaches, mainly including solvent-antisolvent precipitation (conventional in bulk or with the aid of microfluidics), supercritical fluid precipitation and solvent evaporation (spray drying or freeze drying), precisely control precipitation and crystallization of drugs to achieve desired nanosized particles [21]. Bottom-up approaches are favorable in terms of small and narrow-sized distributed particles, but time is needed to establish the suitable conditions, and poor reproducibility barricades scale-up production [15]. In addition, residual organic solvent may yield toxicity, and these approaches are not appropriate for drugs poorly soluble in both aqueous and organic solvents [15]. Considering the pros and cons of top-down and bottom-up approaches, combination approaches sequentially integrate both sides. There have been several successfully developed techniques such as Nanoedge^®^, SmartCrystal^®^ and Nanopure^®^ [22]. For instance, friable crystallized particles with decreased sizes can be preliminarily obtained by solvent-antisolvent precipitation, followed by nanocrystal preparation by high pressure homogenization, which helps to improve the efficiency of particle size reduction for the purpose of easy scale-up, and also avoid clogging of equipment [22]. Nevertheless, due to the increased steps and costs, the necessity of combination approaches needs evaluating before utilization.

### 2.2. Characterization Methods

To substantiate the successful preparation of drug NCs, characterization with various purposes are required, including particle size and distribution, surface properties, morphology, solid state properties, stability and dissolution/drug release rate [23].

Particle size and distribution is commonly measured by dynamic light scattering (DLS), which is fast and repeatable to show basic information of drug NCs that directly affects saturation solubility, physical stability and dissolution/drug release rate [21]. Zeta potential, also measured by DLS, is used to assess surface charge of drug NCs, and it reflects the potential difference between the electric double layer of particles moving under electric field and the layer of dispersant around them at the slipping plane [24]. The shape and size of drug NCs can be observed by scanning electron microscopy (SEM), transmission electron microscopy (TEM) or atomic force microscopy (AFM). Imaging techniques are laborious and time-consuming with only part of particles analyzed, so they are not the first choice for particle size analysis and are usually combined with DLS [23]. The size estimated by TEM should be smaller than that by DLS due to the absence of hydration layer after sample preparation [25].

Solid state form is a key factor for the delivery of poorly water-soluble drugs. Water acts as a plasticizer to stabilize drug crystallinity in the preparation of drug NCs by top-down approaches, while the amorphous form is more typical in bottom-up approaches, and the presence of thermodynamically less stable form might lead to unwanted changes in solid state form during storage, shipping and administration that alter the performance of the system solubility and dissolution rate, diminishing the therapeutic efficacy [23]. The crystalline state of drug NCs are mostly evaluated by X-ray powder diffraction (XRPD) and thermal analysis such as differential scanning calorimetry (DSC). Spectroscopic techniques such as Raman spectroscopy and Fourier transform infrared (FT-IR) spectroscopy are utilized to monitor the crystallization process and to detect the interactions between the drug and excipients, respectively [5].

Physical stability is usually evaluated in phosphate buffer saline (PBS) or fetal bovine serum (FBS), with particle size and surface charge measured by DLS and absorbance by enzyme labeling instrument, respectively [26,27]. Chemical stability, specifically the purity and degradation of drugs, is detected by high performance liquid chromatography (HPLC) that is a most standard way [23].

Dissolution/drug release rate, which can be evaluated by paddle method, dialysis and reverse dialysis sac methods, continuous flow-through cell method and flow-nuclear magnetic resonance (NMR) spectroscopy, reflects the quality of drug NCs and is indispensable for establishing an in vitro-in vivo correlation (IVIVC) [23,28,29]. The dissolution/drug release rate is greatly dependent on factors such as particle size and stabilizers. The increased dissolution rate of drug NCs as compared to bulk drugs is mainly based on larger specific surface area, and according to Noyes-Whitney equation, 100-fold increase in dissolution rate can be achieved if the particle size is reduced from 50 μm to 500 nm [30]. Suitable stabilizers also confer beneficial effects on the release kinetics of the incorporated drug, and drugs with high enthalpy and hydrophobicity tend to show better stability, which can be stabilized by different types of stabilizers [31,32].

## 3. Stability of Drug Nanocrystals

Drug NCs have a smaller particle size than bulk drugs, contributing to increased solubility, dissolution rate and bioavailability and facilitating penetration of drugs through biological barriers inside human body to get to the place where the lesions occur. However, those NCs are endowed with high interfacial free energy by large specific surface area due to reduced particle size so that they are thermodynamically unstable and tend to aggregate [5,23]. Additionally, Ostwald ripening has been frequently observed in NCs growth systems, which is described as a phenomenon that crystalline state constantly changes and smaller particles gradually dissolve and redeposit onto larger ones at supersaturation, resulting in the increase in particle size and poor physical stability such as attraction and aggregation, and thus vascular blockage and embolism after intravenous injection and shortened circulation in blood [4,33,34].

Good stability ensures efficacy as well as safety. In order to improve the stability of drug NCs, suitable stabilizers are carefully selected so as to reduce interfacial free energy and prevent NCs aggregation [15]. Stabilizers also play an important role in further formulation and dramatically affect bioavailability [35,36]. Specifically, stabilizers can be divided into ionic (charged) surfactants, non-ionic surfactants, polymeric stabilizers and other types [32]. Ionic surfactants are represented by sodium dodecyl sulfate (SDS) [37,38,39,40]. Non-ionic surfactants include Tween [41,42,43,44], D-α-tocopherol polyethylene glycol 1000 succinate (TPGS) [27,37] and decyl glycoside [45]. Common polymeric stabilizers are hydroxypropyl methylcellulose (HPMC) [38,40,46,47], sodium carboxymethyl cellulose (CMC-Na) [37], polyvinyl alcohol (PVA) [37,47], polyvinyl pyrrolidone (PVP) [37,47,48], polyethylene glycol (PEG) [43,48] and Poloxamer [41,43,49,50,51,52,53]. There are also some novel stabilizers such as saponins derived from natural plants, which cause less irritation and toxicity in human body as compared to the above chemically synthesized stabilizers [54,55,56].

Generally, ionic (charged) surfactants offer electrostatic stabilization by enhancing repulsive Coulomb forces between particles, whereas non-ionic surfactants and polymeric stabilizers provide steric hindrance stabilization by adsorbing onto particle surfaces and forming a steric layer between particles [32,57]. Not only the increased surface area of particles but also the increased solubility of a drug might contribute to higher dissolution rate of drug NCs formulations, so the stabilizer exhibiting solubility- and dissolution-improving properties is more optimal in this case [41]. Molecular weight (MW) is regarded as another important factor when selecting suitable stabilizers. The optimum concentrations of different polymeric stabilizers are inversely proportional to their molecular weights [47], and faster mucin diffusion and oral absorption can be achieved using polymeric stabilizers of lower molecular weights [40]. Extra mechanisms are also taken into account in stabilizer selection. PEGylation renders drug NCs stealth so as to decrease the uptake by reticuloendothelial system (RES), prolong systemic retention and decrease degradation by enzymes [58,59]. The connection between PEG chains and drug NCs by covalent bonds can lead to improved stability under storage and physiological conditions and enhanced antitumor efficacy [48]. However, it should be noted that when intravenously administered for the first time, PEGylated nanomedicines are recognized as foreign particles by the immune system, leading to the production of anti-PEG antibodies that will accelerate blood clearance by complement activation after their adsorption on the nanosurface (ABC phenomenon) [60]. TPGS, a water-soluble derivative of natural vitamin E, has been reported to show the inhibitory effect of p-glycoprotein (p-gp) efflux that is associated with multidrug resistance (MDR) in cancer chemotherapy [61]. Drug NCs prepared in TPGS have been demonstrated to exhibit sustained release kinetics and achieve better efficacy in chemotherapy-resistant cancer [62].

## 4. Strategies for Active Tumor-Targeted Delivery of Drug Nanocrystals

### 4.1. Characteristics and Limitations of Passive Tumor-Targeted Drug Delivery

The rapid tumor growth requires abundant nutrients supplied through blood vessels, but the hyperpermeable tumor vasculature is completely different from normal blood vessels, which is structurally characterized by lack of basement membranes, morphological abnormalities in pericytes, and low and loose coverage of endothelial cells by pericytes as compared to the tight junction between normal vascular linings [11,63]. The large endothelial pores (10–1000 nm in diameter) in the vessel wall allow large particles (approximately 20–200 nm in diameter) extravasate and accumulate in tumor interstitial spaces, and the absence of functional lymphatic vessels responsible for drainage adds to particle entrapment and retention, which is well known as the EPR effect in solid tumors [10,63]. Enlightened by the features of EPR effect enabling the greater accumulation of nanocarriers in tumors than in normal tissues, a large number of long-circulating nanomedicines with different nanocarriers have been developed and clinically used for the treatment of various malignancies, such as Doxil/Caelyx^®^ (doxorubicin-loaded PEGylated liposomes), Genexol-PM^®^ (paclitaxel-loaded polymeric micelles) and Abraxane^®^ (albumin-bound paclitaxel nanoparticles) [10,12].

Although the EPR effect has long been regarded as the basis for tumor-targeted drug delivery, with evidence showing that the above drug-loaded nanocarriers improve therapeutic efficacy in tumor-bearing murine models [64,65,66], limited clinical benefits are achieved, thus arousing considerable controversy about the EPR effect that it is actually weak and heterogeneous in the setting of human tumors [10]. Substantially, human tumors grow more slowly as compared to established murine tumors, and distribution of pore sizes in tumor vessels, blood flow through tissues, pericyte coverage, basement membrane morphology and extracellular matrix (ECM) density in the tumor microenvironment (TME) of human tumors present high heterogeneity [10]. This might explain the reduced clinical efficacy of nanomedicines despite encouraging results from preclinical studies.

### 4.2. Advantages of Active Tumor-Targeted Drug Delivery

Active tumor-targeted drug delivery often refers to a strategy that ligands or molecules such as proteins, nucleic acids, polysaccharides, peptides, and antibodies, with the ability to specifically target surface receptors highly expressed at targeted sites (tumor endothelial cells, tumor cells or both of them), are modified onto the surface of nanomedicines [12,67]. The enhanced ability to cross biological barriers and efficiency of drug delivery into tumor tissues can be achieved via specific recognition, binding and internalization, thereby increasing drug accumulation at tumor site to improve efficacy, and simultaneously decreasing drug distribution in normal tissues to reduce toxicity [12,67]. There is also an organelle-specific targeting strategy with the intracellular sites including cytoplasm or subcellular organelles such as mitochondria or nucleus [11]. Nature-inspired biomimetic nanocarriers such as cell membrane-coated nanoparticles, which can elevate biocompatibility and prolong circulation, are endowed with active targeting ability due to some surface molecules on the cell membrane that bear the resemblance to modified ligands in terms of tumor-targeted ability [13]. For instance, P-selectin, a surface molecule on platelet membrane, can bind to CD44 expressed on the surface of tumor cells, and homotypic targeting enabled by cancer cell membrane coating is ascribed to surface markers such as Thomsen–Friedenreich antigen and E-cadherin [68]. Generally, surface modification is not restricted by the biological characteristics of cell membranes so that it is considered a promising strategy with broader applications.

**Figure 1:**
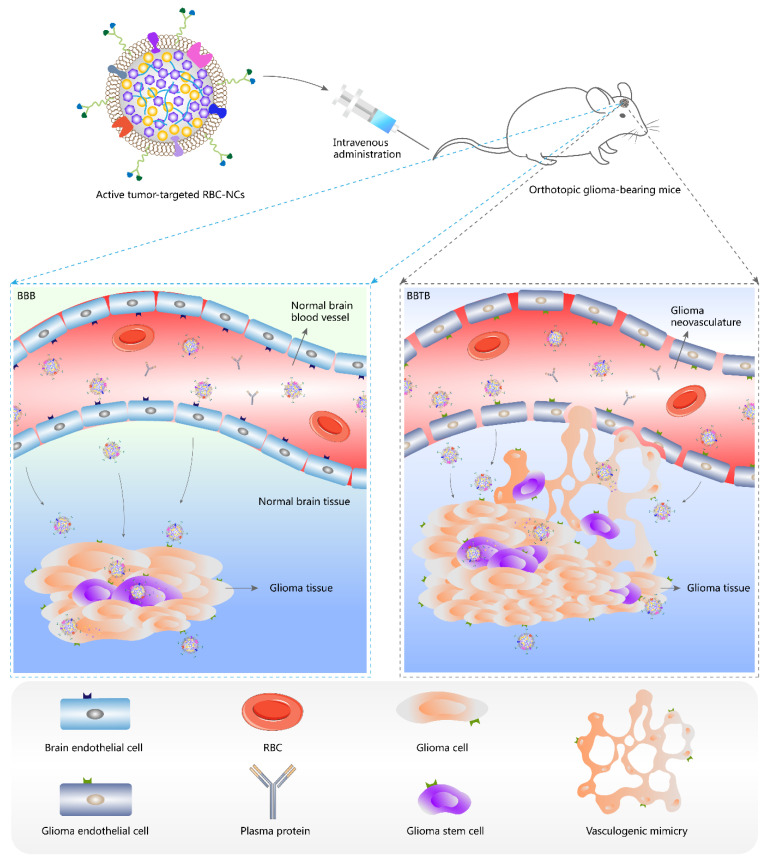
Schematic illustration of active tumor-targeted drug NCs that penetrate BBB and BBTB, and target glioma in vivo. BBB, blood-brain barrier; BBTB, blood-brain tumor barrier; NCs, nanocrystals; RBC, red blood cell; RBC-NCs, red blood cell membrane-coated drug nanocrystals.

**Figure 2:**
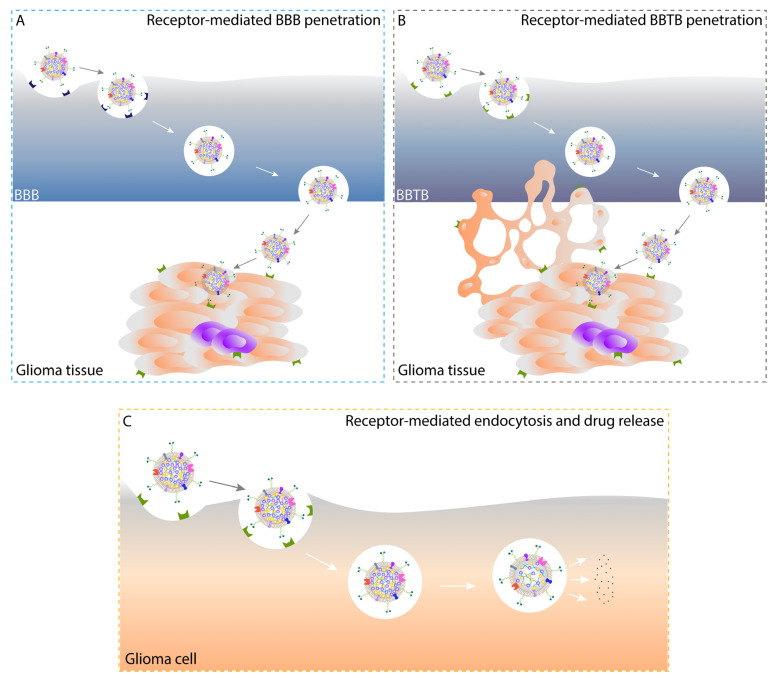
Schematic illustration of receptor-mediated BBB (**A**) and BBTB (**B**) penetration, and receptor-mediated endocytosis and subsequent intracellular drug release (**C**) enabled by active tumor-targeted drug NCs in vivo. BBB, blood-brain barrier; BBTB, blood-brain tumor barrier; NCs, nanocrystals.

Passive targeting facilitates accumulation of nanomedicines in tumor interstitium, but limited internalization by tumor cells is achieved, and furthermore nonspecific uptake by RES might prevent nanomedicines from targeting specific organs (except liver and spleen) or lesion sites [11]. On the contrary, receptor-mediated endocytosis, a typical active targeting enabled by chemically conjugated or physically attached tumor-targeted moieties, is a process consisting of specific binding to receptors on tumor cell surface and subsequent cellular uptake, and intracellular drug release from drug-loaded nanoparticles occurs following their capture in endosomes and arrival at lysosomes or other subcellular organelles, which can precisely ensure the therapeutic concentrations at targeted sites [11]. For brain malignancies such as glioma, conventional chemotherapy has failed due to the poor ability to cross the EPR-weak blood-brain barrier (BBB) that is physiologically responsible for the maintenance of homeostasis in the brain [69,70,71]. The newly formed blood-brain tumor barrier (BBTB) with disease progression, structurally manifesting as relatively narrow fenestrae in glioma neovasculature, permits minimal paracellular and transcellular transport of particles [72,73]. The existence of glioma stem cells (GSCs) and vasculogenic mimicry (VM) formed by invasive glioma cells differentiated from GSCs adds to drug resistance and recurrence of glioma [74,75]. In this case, surface modification of nanocarriers with multifunctional targeting ligands, to a large extent, enhances the penetration of drug-loaded nanoparticles through BBB and BBTB, eradicates glioma cells and GSCs, and destructs glioma neovasculature and VM as well, indicating the potential of a novel all-stage precision tumor-targeted therapy (Figure 1 and Figure 2) [76].

In a nutshell, active tumor-targeted drug delivery shows advantages of accurate drug biodistribution and thus reduced administration dosage, as well as biological-barrier penetration over passive targeting, leading to good efficacy and acceptable safety.

### 4.3. Factors Influencing Therapeutic Efficacy of Drug Nanocrystals

Shape and size are two key factors that determine physicochemical, pharmacokinetic as well as pharmacodynamic properties of drug NCs. Benefits seem to be achieved from drug NCs with a shape of high aspect ratio such as rod/needle [77]. 10-hydroxycamptothecin (HCPT) NCs were prepared by supercritical anti-solvent technique combined with high pressure homogenization, and after the bottom-up process, the crystalline shape of HCPT changed from a pancake-like form to prismatic and needle-like forms, and further top-down process had particle size-reducing but shape-maintaining effects on HCPT NCs. Among the three forms, needle-shaped HCPT NCs with controllable particle size and large specific surface area showed dramatically improved antitumor efficacy by intravenous route, which might be attributed to longer blood retention time resulting from high saturation concentration and sustained release behavior, and more efficient cellular uptake benefiting from the uniformly distributed particle size, specific morphology and polymorph of NCs. Lower systemic toxicity was observed with needle-shaped HCPT NCs than with commercial HCPT injections [78]. In oral drug delivery, rod-shaped NCs, as compared to sphere- and flake-shaped ones, greatly improved absorption efficiency due to enhanced mucus permeation, cellular uptake and transepithelial transport [79]. Larger specific surface area of rod shape than that of sphere shape probably facilitates ligand modification onto drug NCs surface in a larger number [80]. Intriguingly, compared with sphere-shaped HCPT NCs, the rod-shaped counterpart exhibited better antitumor efficacy, but simultaneously caused higher systemic toxicity, which calls for balanced consideration of both efficacy and safety of drug NCs formulations in future investigation [81]. Besides, in two shapes with three dimensions of comparable length [77], cube-shaped theranostic paclitaxel (PTX) prodrug NCs with surface functionalization showed superiority over the sphere-shaped counterpart in sustained release behavior, improved cellular uptake efficiency and enhanced cytotoxicity in human cervical carcinoma cell line HeLa, suggesting the feasibility of its application in both imaging and chemotherapy [82].

For liposomal drug NCs formulations, different shapes and sizes can be achieved by varying lipid bilayer compositions and drug-to-lipid ratio, leading to controllable drug release behavior [83,84]. PEGylated liposomes were sphere-shaped before drug crystallization, whereas they were stretched to be rod-shaped by drug NCs after freeze-thawing, providing a great opportunity to modulate drug release behavior and develop high drug-loaded formulations [85]. For local injectable hydrogels loaded with different sized particles, rod-shaped PTX NCs with intermediate particle size (about 120 nm) was optimal owing to high drug loading and moderate drug release rate, thereby leading to preferable antitumor efficacy, while PTX microcrystals (over 2 μm) exhibited high drug loading but minimal drug release, and PTX micelles (about 20 nm) demonstrated excessively quick drug release and thus insufficient local drug concentration [86].

### 4.4. Application of Drug Nanocrystals in Active Tumor-Targeted Drug Delivery

The efficacy and safety of active tumor-targeted drug NCs delivery systems have been greatly investigated and discussed both in vitro and in vivo, yielding a large number of valuable results for reference. Generally, there are two types of active targeting strategies: one is the EPR utilization and tumor cell-based active targeting evaluated in the subcutaneous tumor-bearing animal model, and the other is biological-barrier crossing- and tumor cell-based active targeting evaluated in the orthotopic tumor-bearing animal model (Table 2).

#### 4.4.1. EPR Utilization and Tumor Cell-Based Active Targeting

i. Folic acid conjugate

The term folate encompasses both natural folates and folic acid (FA, also known as water-soluble vitamin B9), with the latter employed to signify the fully oxidized chemical compound constituting an important source of ingested folates through supplements [87]. Folate receptor (FR) family are glycoproteins with high affinity for FA, and among the three functional isoforms, FR_α_ that is attached to cell membrane through a glycosylphosphatidylinositol (GPI) anchor is the most commonly expressed one, which is overexpressed in a variety of epithelial cancers but restrictedly expressed in normal tissues [11]. In addition to targeting ability, conjugation of FA shows advantages including functional stability in preparation, good biocompatibility, and lack of immunogenicity [87].

FA-conjugated PTX NCs (PTX/F127-folate) resulted in significantly higher cytotoxicity in the FR-positive human epithelial carcinoma cell line KB than unconjugated PTX NCs at the concentrations ranging from 0.08 to 10 μM [88]. PIK-75, a phosphatidylinositol 3-kinase (PI3K) inhibitor, has shown antitumor activity in vitro and in vivo, but it lacks solubility and stability, with the likelihood of off-target toxicity in normal tissues. In human ovarian cancer cell line SK-OV-3, FA-conjugated PIK-75 NC suspension (PIK-75-NS-FA) exhibited increased cellular uptake and cytotoxicity as compared to the unconjugated counterpart. In subcutaneous SK-OV-3 tumor-bearing mice, improved bioavailability and increased PIK-75 accumulation in tumor were observed with PIK-75-NS-FA. Enhanced downregulation of phospho-Akt in tumor tissues also indicated the enhanced drug delivery efficiency and cytotoxicity by active-targeting treatment [89]. Dopamine can self-polymerize and form a tight polydopamine (PDA) layer on the surface of solid materials in weak basic environment so as to provide a versatile platform for reaction with thiol or amine groups at *o*-quinone moieties, benefiting diverse surface functionalization [90,91,92,93]. Surface-modified FA-conjugated PDA facilitated selective cellular internalization of zein-based nanocomplexes encapsulated with needle-shaped HCPT NCs (HCPT@AuNPs-Zein-PFA) and thus enhanced endocytosis, with better antitumor activity and lower toxicity as compared to the non-targeting equivalent in vitro and in vivo [94]. Lipid layers of liposome contribute to high stability and easy surface functionalization. A hydrophobic drug candidate with the potent inhibitory effect on chronic myeloid leukemia (CML) was selected as a model drug, and the prepared core-shell FA-functionalized NC-loaded liposome (053-NC@FA-Lipo) exhibited higher cellular uptake efficiency than the surface PEGylated formulation in FR-overexpressed human CML cell lines K562 and KU812. Furthermore, compared with free drug, intravenously administered 053-NC@FA-Lipo led to better antitumor efficacy in subcutaneous K562 tumor-bearing mice at a lower dosage, suggesting the potential for the development of this nano-in-nano delivery system [95].

ii. Transferrin conjugate

Transferrin (Tf) is a glycoprotein that is important in iron metabolism, by which iron is safely carried in blood circulation and delivered to different tissues such as liver and spleen [11]. Tf specifically binds to Tf receptors (TfR) that are extremely overexpressed in cancer cells with increased iron demand for rapid proliferation [96].

Compared with free docetaxel (DTX), both Tf-modified DTX NCs (Tf-DTX-NCs) and unmodified DTX NCs displayed rapid drug release behavior, but Tf-DTX-NCs showed significantly higher cellular uptake for 2-h incubation and higher toxicity for 72-h incubation than DTX NCs in human non-small cell lung cancer (NSCLC) cell line A549 [97]. Similar encouraging results were found by comparison between Tf-modified PTX NCs (Tf-PTX-NC) and PTX NCs in human breast cancer cell line MCF-7, and additionally, Tf-PTX-NCs had lower toxicity in immortalized human keratinocyte cell line HaCaT, indicating benefits in both efficacy and safety [98]. In addition to the TfR-targeting ability, Tf was also found to act as a natural stabilizer in Tf-modified PTX NCs (PTX-Tf), which demonstrated good stability over at least 3 months at 4 °C, higher tumor inhibition rate than the unmodified counterpart, and lower toxicity than Taxol^®^ [99]. Modulation of exposed NC facets could enhance specific NC-Tf association via inner-sphere coordination in a complex protein matrix, thus improving TfR-mediated delivery of drug NCs into cancer cells [100].

iii. Hyaluronic acid conjugate

Hyaluronic acid (HA) is a natural polysaccharide made up of glucuronic acid and *N*-acetylglucosamine, which can be utilized as a sustained-release carrier for drugs ascribed to its high viscoelasticity, plasticity together with nonimmunogenicity, good biocompatibility and degradability [101]. HA has also demonstrated tumor-targeting ability due to its high affinity for CD44, a family of single-pass transmembrane glycoproteins overexpressed in a variety of cancer cells and recognized as a surface marker for cancer stem cells (CSCs) [11,102].

HA-modified PTX-NCs (HA-PTX-NC) showed faster drug release, higher cellular uptake efficiency and stronger inhibitory effect in CD44-expressed cancer cells as well as lower toxicity in normal cells as compared to unmodified PTX-NCs [98]. Another study reported that rod- or needle-shaped HA-conjugated camptothecin (CPT) NCs (HA-CPT-NC) exhibited improved aqueous dispersion, enhanced stability and prolonged circulation owing to nanoscaled particle size and hydrophilic HA layer. After HA-CPT-NC treatment, stronger cytotoxicity and apoptosis were observed in CD44-overexpressed human breast cancer cell line MDA-MB-231, lower toxicity in human fetal lung fibroblast cell line IMR-90, and favorable biocompatibility in mice. Mechanistically, specific HA-CD44 binding triggered receptor-mediated endocytosis and intracellular mitochondria-mediated apoptosis [103].

iv. Albumin conjugate

Albumin is the most abundant plasma protein involved in the transendothelial transport of nutrients or drugs with low toxicity, biocompatibility and the ability to reduce opsonization and phagocytosis, and rapidly proliferating cancer cells have an increased ability to take up albumin because of increased demand for nutrients and energy [104,105]. Uptake of albumin conjugates are presumably mediated by gp60 transcytosis pathway and subsequent specific binding to secreted protein acidic and rich in cysteine (SPARC) in tumor interstitium, and overexpression of SPARC is associated with enhanced tumor invasion, metastasis and thus poor prognosis [106].

The formulation of albumin-conjugated PTX NCs crystalized in F127 (Cim-F-alb) had the smallest particle size and the most native albumin as compared to that in hexadecyltrimethylammonium bromide (CTAB) and pure NCs. Cim-F-alb showed better serum stability than Abraxane^®^, and high cellular uptake efficiency, comparable cytotoxicity to that of Abraxane^®^ in the SPARC-positive murine melanoma cell line B16F10. Cim-F-alb also demonstrated higher antitumor efficacy than Abraxane^®^ in subcutaneous B16F10 tumor-bearing mice at the same dosage [104]. Further analysis on pharmacokinetics and biodistribution in vivo added that Cim-F-alb exhibited prolonged circulation time and higher tumor accumulation than Abraxane^®^, and less interaction with serum proteins were ascribed to albumin coating [107]. Similarly, stable sheet-shaped albumin-conjugated DTX NCs were successfully prepared using F127 as the stabilizer (DTX-F-alb), and increased cellular uptake of DTX-F-alb versus free DTX was observed in multidrug-resistant human ovarian cell line NCI/ADR-RES highly expressing SPARC, which could be attenuated by SPARC knockdown [108].

v. Chondroitin sulfate conjugate

Chondroitin sulfate (CS) is a natural polysaccharide belonging to sulfated glycosaminoglycan (GAG) that has been found in ECM, corneas, skin and cartilage, showing good compatibility, and similar to HA, receptor-mediated endocytosis can be triggered by CS specifically binding to CD44 [109].

CS-conjugated DTX NCs (DTX@CSA-NCs) outperformed the unconjugated counterpart and Taxotere^®^ (DTX injection) as evidenced by enhanced cellular uptake together with higher degree of cytotoxicity and G2-phase arrest in a time-dependent manner in MDA-MB-231 cells, mechanistically due to disrupted mitochondrial membrane potential and lysosomal membrane integrity. Furthermore, intravenously administered DTX@CSA-NCs showed higher plasma concentration in healthy SD rats and stronger tumor inhibition in subcutaneous 4T1 tumor-bearing mice [110]. Increased level of hyaluronidase (HAase) in various malignancies will rapidly cleave linkages between alternating disaccharide units in CS chain in acid environment and lead to subsequent CS degradation into low molecular weight fragments, providing more gaps on the particle surface for drug diffusion with little disruption of the interaction and attachment of a CS derivative (CSOA) to doxorubicin (DOX) NCs (CSOA/NCs). The prepared CSOA/NCs, with a sphere shape and particle size of about 102 nm, exhibited accelerated drug release behavior in response to HAase or incubated with cancer cells, and high cellular uptake of CSOA/NCs was observed in CD44-overexpressed cancer cells rather than CD44-negative normal fibroblasts [111].

vi. Mannuronic acid conjugate

D-mannuronic acid (MA) is an important component of a natural polysaccharide alginate that can specifically bind to mannose receptor (MR) mostly expressed by macrophages, dendritic cells and endothelial cells, and MR is also found overexpressed in many cancer cells because of their higher affinity for carbohydrate molecules than that of normal cells due to higher demand for nutrients [112]. 

A cytosolic co-delivery system of immunological adjuvant Poly I:C and PTX NCs coated with MA (MA-PNRplex), which was rod-shaped with particle size of about 218 nm, greatly enabled immunogenicity of cancer cells as evidenced by increased secretion of cytokines and chemokines and enhanced immune response in vivo, leading to improved antitumor efficacy. Specifically, the intracellular asynchronous release of Poly I:C and PTX amplified antitumor immune response [113].

vii. Herceptin conjugate

The human epidermal growth factor receptor (HER) family are transmembrane glycoproteins associated with pathogenesis of various cancers, and HER2 has been found overexpressed in approximately 20% of all breast cancers, which is characterized by activation of downstream signaling after dimerization [11,114]. Herceptin^®^ (HCT, trastuzumab) is a humanized monoclonal antibody targeting HER2, and has been approved by the U.S. Food and Drug Administration (FDA) as monotherapy or combination with chemotherapy for the treatment of early-stage HER2-positive breast cancer or metastatic breast cancer [115].

Rod-shaped HCT-conjugated PTX NCs (PNCs-HCT) were stable for at least 1 month at 4 °C and displayed similar sustained drug release behavior to unconjugated PTX NCs. Higher binding affinity and cell-specific internalization to HER2-positive cancer cell lines (BT-474, SK-BR-3, and SK-OV-3) with PNCs-HCT than with unconjugated PTX NCs were evidenced by increased cellular uptake and cytotoxicity as well as enhanced cell-cycle arrest in G2/M phase [80]. A similar study revealed that stable HCT-conjugated DTX NCs (HCT-DTX-NCs), with a pebble shape and mean particle size of 542.4 ± 195.0 nm, exhibited drug release behavior in a more rapid way, and increased cellular uptake and cytotoxicity in HER2-positive breast cancer cells than unconjugated DTX NCs [115]. Additionally, PTX NCs self-assembling into worm-like micelles with HCT conjugated on the outside surface showed good antitumor efficacy in subcutaneous SK-BR-3 tumor-bearing mice [116]. Specifically, in active tumor-targeted drug delivery, HCT-HER2 interaction can lead to blockage of receptor cleavage, activation of antibody-dependent cellular cytotoxicity (ADCC) and receptor degradation after endocytosis of HCT-HER2 conjugate [11]. This HCT-conjugated drug NCs strategy greatly exemplifies combination therapy of antibody and chemotherapeutic agents.

viii. Peptide conjugate

Tumor-targeting peptides, usually composed of less than 50 amino acids, are naturally existing or artificially synthesized low molecular weight ligands that are able to specifically bind to receptors expressed on the surface of cells. They have higher cell or tissue permeation ability as compared to proteins or antibodies, improved pharmacokinetic behavior with little attenuated targeting ability by suitable chemical modification, and good stability in vivo owing to resistance to protease hydrolysis [117].

XQ-1 (HAIYPRHGGGF) is a novel tumor-targeting peptide with high affinity for TfR. Rod-shaped PDA-coated CPT NCs with surface modification by XQ-1 exhibited improved stability, dispersion property and dissolution rate, and more importantly, enhanced targeting ability to TfR-overexpressed A549 and HeLa cells and thus cytotoxicity as compared to free CPT and unmodified CPT NCs [90].

Integrins are large heterodimeric transmembrane glycoproteins made up of noncovalently bound α- and β-subunits, and they are critical in cell-to-ECM and cell-to-cell communications and play an active role in signaling pathways concerning cell proliferation, division, and survival [11]. Integrin α_V_β_3_ is found to be overexpressed in tumor cells and neovasculature [118], and RGD (arginine-glycine-aspartic acid) is a well-designed integrin α_V_β_3_-targeting peptide that has been widely used to improve the antitumor efficacy of nanomedicines [119]. Based on PDA coating, a stable near-sphere-shaped PEGylated PTX NCs modified with RGDyc peptide (NC@PDA-PEG-RGD) were successfully prepared. NC@PDA-PEG-RGD showed increased cellular uptake and cytotoxicity in A549 cells, and increased drug accumulation in tumor and enhanced antitumor efficacy in subcutaneous A549 tumor-bearing mice as compared to unmodified PEGylated PTX NCs [92]. NR1 (RGDARF) is another tumor-targeting hexapeptide containing RGD motif so that it can also specifically interact with integrin α_V_β_3_. NR1-modified inorganic silver nanoparticles (AgNP)-decorated PTX NCs showed dramatically improved cellular uptake efficiency and cytotoxicity in MDA-MB-231 cells [120].

ix. Triphenylphosphonium cation conjugate

Mitochondria are novel targets for treating cancer, especially in the setting of MDR, and triphenylphosphonium cation (TPP^+^) is a mitochondrial homing moiety with high lipophilicity. TPP^+^-modified PTX NCs were reported to show the strongest cytotoxicity against 2D monolayer of MCF-7 and MCF-7/ADR cells as well as deeper penetration on 3D multicellular spheroids (MCs) of MCF-7 cells and more potent growth inhibition on MCF-7 and MCF-7/ADR MCs, providing a promising therapeutic option to overcome drug-resistant breast cancer [121].

x. Cell membrane-coated formulation

Circulating tumor cells (CTCs) derived from local tumors are capable of escaping immunosurveillance and targeting homotypic tumors due to cell surface interactions including Thomsen–Friedenreich antigen and E-cadherin, and inspired by this self-adherence property, researchers have developed the biomimetic cancer cell membrane-coating technique for active tumor-targeted drug delivery [13,68]. 

Flake-shaped 4T1 cancer cell membrane-coated HCPT NCs sandwiched with a photosensitizer indocyanine green (NCs/ICG/CM) greatly induced effective uptake by 4T1 cells rather than HeLa cells, human umbilical vein endothelial cells (HUVEC) or murine macrophages J774A.1, and showed enhanced antitumor efficacy with little toxicity in subcutaneous 4T1 tumor-bearing mice, which provides a promising modality for utilization of hydrophobic chemotherapeutic agents in cancer treatment [122].

#### 4.4.2. Biological-Barrier Crossing- and Tumor Cell-Based Active Targeting

i. Albumin conjugate

Albumin facilitates drug permeation across tumor vessels. Compared with current cyclodextrin-based formulation that are limited by short circulation time of carfilzomib (CFZ) due to extensive hepatic and extra-hepatic metabolism and nonspecific biodistribution, a novel albumin-coated CFZ NCs (CFZ-alb NC) had advantages of improved metabolic stability, enhanced cellular uptake and cytotoxicity in different breast cancer cell lines (human: MDA-MB-231, HCC1143 and HCC1937; murine: 4T1), and greater antitumor efficacy in orthotopic 4T1 tumor-bearing mice owing to the improved ability to cross the blood-tumor barrier (BTB) and then target tumor cells [105].

ii. Peptide conjugate

As mentioned before, constant changes in crystalline state during NCs growth will be detrimental to dosing, especially via intravenous route [4]. It has been increasingly validated that despite high stability and easy surface functionalization, lipid membrane-coated nanocarriers such as liposomes are prone to adsorb natural IgM upon entry into blood to form protein corona, thereby resulting in ABC phenomenon [123,124]. Red blood cell (RBC) has the advantages of good biocompatibility and long circulation in blood [13], and RBC membrane coating helps to prolong systemic circulation and avoid ABC of nanoparticles by the immune system after multiple doses [125]. RBC membrane-coated DTX NCs modified with cyclic RGDyK (c(RGDyK)) peptide via a facile insertion method involving multivalent avidin-biotin interactions (RGD-RBC-NC(DTX)) displayed a core-shell structure with particle size of about 70 nm, and demonstrated improved long-term stability, excellent biocompatibility, and prolonged retention time. Superior tumor accumulation and therapeutic efficacy in both subcutaneous and intracranial U87 glioma-bearing mice benefited from the ability to penetrate BBTB and target tumor cells mediated by the interaction between c(RGDyK) and integrin α_V_β_3_ [26].

iii. Cell membrane-coated formulation

In the absence of targeting ligand modification, HCPT NCs coated with cancer cell membrane from rat glioma cell line C6 (HCPT-NS/CCM) still had the ability to penetrate BBB and BBTB, and target glioma via immune escape and homotypic binding as evidenced by enhanced BBB and BBTB permeability, increased cellular uptake by C6 cells and increased glioma accumulation in intracranial C6 tumor-bearing mice when compared to HCPT-NS [126].

iv. Peptide-conjugated cell membrane-coated formulation

To further the targeting ability, the strategy of tumor-targeting peptide modification and that of biomimetic cancer cell membrane coating are combined. PTX NCs were coated with C6 cancer cell membrane and then modified with ^D^WSW (^D^S^D^Y^D^P^D^G^D^W^D^S^D^W), a D-peptide exhibiting good proteolytic stability and barrier-penetrating ability [127], to obtain the formulation (^D^WSW-CCM-(PTX)NS). ^D^WSW-CCM-(PTX)NS could effectively penetrate BBB and specifically target glioma tissues, and compared with unmodified CCM-(PTX)NS, pure (PTX)NS and free PTX, ^D^WSW-CCM-(PTX)NS via intravenous administration showed the strongest tumor inhibitory effect and led to longest lifespan in intracranial C6 tumor-bearing mice, suggesting the feasible application of this integrated strategy in the treatment of glioma and probably other cancers [128].

**Table 2:** Current research on active tumor-targeted drug NCs delivery systems conducted both in vitro and in vivo.

Targeting Moieties	Receptor	Model Drug	Stabilizer	Preparation Technique	Particle Size (nm)	Morphology	Evaluation of Efficacy and Safety		References
							In Vitro	In Vivo	
Evaluation in subcutaneous animal models									
FA	FR	PIK-75	F68 and SBL-PC	High pressure homogenization	161 ± 40	Sphere	SK-OV-3 cells	Subcutaneous SK-OV-3 tumor-bearing mice	[89]
FA	FR	HCPT	/	Supercritical antisolvent precipitation followed by ultrasonic dialysis	189.7 ± 9.5	Needle; core-shell after encapsulation into nanocomplex	KB, HeLa and A549 cells	Subcutaneous KB tumor-bearing mice	[94]
FA	FR	053	F127	Wet ball milling followed by ultrasonication	≈183.3	Rod; core-shell after encapsulation into liposomes	K562 and KU812 cells	Subcutaneous K562 tumor-bearing mice	[95]
Tf	TfR	PTX	/	Solvent-antisolvent precipitation	304 ± 13	Rod	SK-OV-3 and KB cells	Subcutaneous KB tumor-bearing mice	[99]
Albumin	SPARC	PTX	F127	Solvent evaporation	196.7 ± 34.6	Rod	B16F10 cells	Subcutaneous B16F10 tumor-bearing mice	[104]
CS	CD44	DTX	PEG and PVP	High pressure homogenization	194 ± 9	Rod	MDA-MB-231, MCF-7 and 4T1 cells	Subcutaneous 4T1 tumor-bearing mice	[110]
MA	MR	Poly I:C and PTX	CLG	Solvent-antisolvent precipitation	≈218	Rod	B16F10 cells	Subcutaneous B16F10 tumor-bearing mice	[113]
HCT	HER2	PTX	PCL-PEG	Solvent evaporation	144 ± 16	Worm-like	SK-BR-3 and MDA-MB-231 cells	Subcutaneous SK-BR-3 tumor-bearing mice	[116]
RGD peptide	Integrin α_V_β_3_	PTX	TPGS and citrate acid	Solvent evaporation	419.9 ± 80.9	Near-sphere	A549 cells	Subcutaneous A549 tumor-bearing mice	[92]
Evaluation in orthotopic animal models									
Albumin	SPARC	CFZ	F127	Solvent evaporation	270.8 ± 21.5	Rod	MDA-MB-231, MCF-7, HCC1143, HCC1937 and 4T1 cells	Orthotopic 4T1 tumor-bearing mice	[105]
RGD peptide	Integrin α_V_β_3_	DTX	F127	Solvent evaporation	≈70	Irregular; core-shell after RBC membrane coating	U87 cells	Subcutaneous and orthotopic U87 tumor-bearing mice	[26]
^D^WSW peptide	QSR	PTX	PVP and SDC	Solvent-antisolvent precipitation	≈169	Sphere; core-shell after C6 cancer cell membrane coating	4T1, B16F10, HepG2 and C6 cells	Orthotopic C6 tumor-bearing mice	[128]

Notes: A549 cells, human non-small cell lung cancer cell line; B16F10 cells, murine melanoma cell line; CFZ, carfilzomib; CLG, cationic β-LG; CS, chondroitin sulfate; C6 cells, rat glioma cell line; DTX, docetaxel; FA, folic acid; FR, folate receptor; F127, Pluronic F127; F68, Pluronic F68; HCC1143 cells, human breast cancer cell line; HCC1937 cells, human breast cancer cell line; HCPT, 10-hydroxycamptothecin; HCT, Herceptin^®^; HeLa cells, human cervical carcinoma cell line; HepG2 cells, human hepatocellular carcinoma cell line; HER2, human epidermal growth factor receptor 2; KB cells, human epithelial carcinoma cell line; KU812 cells, human chronic myeloid leukemia cell line; K562 cells, human chronic myeloid leukemia cell line; MA, D-mannuronic acid; MCF-7 cells, human breast cancer cell line; MDA-MB-231 cells, human breast cancer cell line; MR, mannose receptor; NCs, nanocrystals; PCL-PEG, poly(ε-caprolactone)-*co*-poly(ethylene oxide); PEG, polyethylene glycol; PTX, paclitaxel; PVP, polyvinyl pyrrolidone; QSR, quorum-sensing receptor; RBC, red blood cell; SBL-PC, soybean lecithin with 70% phosphatidylcholine; SDC, sodium deoxycholate; SK-BR-3 cells, human breast cancer cell line; SK-OV-3 cells, human ovarian cancer cell line; SPARC, secreted protein acidic and rich in cysteine; Tf, transferrin; TfR, transferrin receptor; TPGS, D-α-tocopherol polyethylene glycol 1000 succinate; U87 cells, human glioma cell line; 4T1 cells, murine breast cancer cell line.

## 5. Conclusions and Future Perspectives

To overcome the limitations of conventional antitumor pharmaceutical agents, considerable efforts have been made in the delivery of poorly water-soluble drugs using the technology of drug NCs for the advantages of high drug loading, good physical stability as well as improved drug release behavior and bioavailability. Active tumor-targeted strategies such as ligand modification and cell membrane coating not only improve antitumor efficacy but also reduce toxicity ascribed to the ability endowed to penetrate biological barriers and specifically target tumor site. Drug NCs have provided a good nano-platform for the delivery of drugs via oral, intravenous, and other administration routes, and they can also be encapsulated into nanocarriers such as liposomes for better stability. Despite abundant encouraging results and significant progress, some key points should be taken into meticulous consideration, and also some problems warrant further investigation.

Currently, common chemotherapeutic drugs, including classical taxanes PTX and DTX, have been selected as the model drugs in drug NCs formulations for cancer treatment. Cabazitaxel (CTX) is a novel semi-synthetic taxane with poor affinity for drug resistance-related drug efflux pump p-gp and improved BBB-penetrating ability that has shown better antitumor activity than PTX and DTX, but disadvantages such as poor water solubility, toxicity and low selectivity to tumor tissues limit its application [129], which might be well resolved by active-tumor targeted drug NCs delivery systems. In addition to single-agent administration, the delivery of composite NCs of two different drugs generating synergistic antitumor activity at an appropriate ratio can provide another regimen [27]. In the preparation of drug NCs formulations, preparation techniques with high efficiency and good reproducibility will be beneficial to scale-up production of drug NCs with controllable physiochemical properties especially particle size and shape, which will facilitate clinical translation. Suitable stabilizers are selected according to their properties such as molecular weight, functional group, and solubility for drugs, and furthermore, potential formulations with naturally derived stabilizers or even with no stabilizers might exhibit better safety with comparable antitumor efficacy to those with chemically synthesized stabilizers if appropriately designed and prepared. Cutting-edge technology, such as machine learning for the prediction of particle size and distribution [130], a web-based crystallographic tool for the modelling of crystal structure [131], and 3D printing for the production of immediate-release solubility-improved oral polymeric films [132], paves a new way for well controlled preparation and production of drug NCs.

From the perspective of current active targeting strategies, targeted receptors should be highly expressed on tumor endothelial cells and/or tumor cells rather than normal cells so as to prevent off-target toxicity. Ligands with high affinity for targeted receptors are modified onto the surface of drug NCs, and specific ligand-receptor binding triggers receptor-mediated endocytosis for internalization of drug-loaded particles and subsequent intracellular drug release. Distinct from peripheral tumors, the physiologically existing BBB and subsequently formed BBTB are major obstacles to efficient drug delivery for glioma occurring in the brain, so tumor-targeting ligands with multiple functions, such as peptides consisting of BBB- and BBTB-penetrating molecules via a chemical linker, will boost the antitumor efficacy to the fullest extent and ensure safety simultaneously [76]. Suitable approaches of surface modification are requisite for maintaining the stability of drug NCs, and PDA or liposome coating facilitate surface functionalization, but it has been noted that protein corona formed by natural IgM adsorption on the surface of liposomes will lead to ABC phenomenon. Biomimetic cell membrane coating enables improved stability, good biocompatibility and prolonged systemic retention of drug NCs, and further surface modification with targeting ligands effectively circumvents the interference of membrane negative charges on the functions of positively charged targeting ligands as well as the steric hindrance of membrane proteins on the specific binding of targeting ligands [26]. Moreover, to guarantee the integrity of favorable biological functions of cell membrane including long circulation and active targeting after membrane isolation, determination of specific surface markers should be involved.

Other active targeting strategies gaining increasing attractions might be a source of inspiration for the development of novel active tumor-targeted drug NCs delivery systems. Combination of tumor-targeting ligand modification and biomimetic cancer cell membrane coating will enhance the specificity towards tumor cells and/or tissues via ligand-receptor binding and homotypic binding. An integrated strategy of surface modification with tumor-targeting peptide and cell-penetrating peptide (CPP) conjugate will contribute to improved antitumor efficacy due to dual benefits from specific targeting at tumor site and enhanced internalization across cell membranes [93,133,134]. More microscopically, organelle-specific targeting is an emerging strategy with the advantages of precise optimization of dosing parameters in organelles such as mitochondria, nucleus and lysosome and prevention of possible toxicity to organelles and other subcellular structures arising from arbitrary drug incorporation inside cytoplasm [11]. Besides, biomimetic drug delivery strategies such as living cell vehicles enable lung-metastasis targeting and controlled drug release [135,136]. Notably, attention has been paid to the immune system other than tumor itself since tumor-associated macrophages (TAMs) infiltrating the surrounding tumor tissues in TME play a crucial role in tumor occurrence, progression and metastasis. Based on the antitumor effect of M1 macrophages and the tumorigenic effect of M2 macrophages, precise M2 TAM-targeted delivery system enabled by integrated active-escape and active-targeting signals, which was validated to enhance the potency of antitumor therapy, has also provided a new option [137].

Accumulating evidence has supported the potential of drug NCs for active tumor-targeted drug delivery, but it should be aware that the subcutaneous tumor-bearing animal models, which have been broadly used in current studies, are actually not that appropriate because of the augmented EPR effect as compared to that in human tumors clinically. Therefore, more attention should be paid to the utilization and improvement of orthotopic tumor-bearing animal models that take into account efficient penetration through biological barriers such as BBB, BTB and BBTB. This is an important issue for active targeting in addition to receptor-mediated endocytosis and will lay the foundation for substantial clinical evaluation in the future to take a further step from bench to bedside. In this seemingly endless fight against cancer, we sincerely hope that more and more safe and efficacious nanomedicines, represented by active tumor-targeted drug NCs, will be developed and eventually succeed in clinical translation.

## Figures and Tables

**Table 1:** Typical marketed drug NCs products manufactured by top-down approaches.

Drug (Brand Name and Company)	Category	Manufacturing Technique	Dosage Form	FDA Approval Year	Major Indication	References
Oral route						
Sirolimus (Rapamune^®^, Pfizer/Wyeth)	Immunosuppressant	Media milling	Tablets	2000	Prevention of organ rejection in renal transplantation	[15,16,17]
Aprepitant (Emend^®^, Merck)	Antiemetic	Media milling	Capsules	2003	Prevention of nausea and vomiting caused by chemotherapy	[15,16,17]
Fenofibrate (TriCor^®^, Abbott)	Hypolipidemic agent	Media milling	Tablets	2004	Treatment of hyperlipoproteinemia	[15,16,17]
Megestrol acetate (Megace^®^ ES, Par Pharmaceutical)	Progestin	Media milling	Suspension	2005	Treatment of anorexia and cachexia, or unexplained, significant weight loss in patients with AIDS	[15,16,17]
Naproxen sodium (Naprelan^®^, Pfizer/Wyeth)	Nonsteroidal anti-inflammatory drug	Media milling	Tablets	2006	Treatment of pain or inflammation caused by arthritis, ankylosing spondylitis, etc.	[15]
Theophylline (Theodur^®^, Mitsubishi Tanabe Pharma)	Bronchodilator	Media milling	Tablets	2008	Treatment of asthma and bronchitis	[15]
Fenofibrate (Triglide^®^, Skyepharma)	Hypolipidemic agent	High pressure homogenization	Tablets	2005	Treatment of hyperlipoproteinemia	[15,16,17]
Intravenous route						
Meloxicam (Anjeso^®^, Baudax Bio)	Nonsteroidal anti-inflammatory drug	Media milling	Suspension	2020	Treatment of moderate to severe pain	[18]
Cabotegravir and rilpivirine (Cabenuva^®^, ViiV Healthcare)	Antiviral combinations	Media milling	Suspension	2021	Treatment of AIDS	[19]
Paliperidone palmitate (Invega Sustenna^®^, Johnson & Johnson/Janssen)	Atypical antipsychotic	High pressure homogenization	Suspension	2009	Treatment of schizophrenia	[15]
Aripiprazole lauroxil (Aristada^®^, Alkermes)	Atypical antipsychotic	High pressure homogenization	Suspension	2015	Treatment of schizophrenia	[20]

Notes: AIDS, acquired immune deficiency syndrome; FDA, the U.S. Food and Drug Administration; NCs, nanocrystals.

## Data Availability

Not applicable.
